# Osteoporosis and Fracture Risk Following Benign Hysterectomy Among Female Patients in Korea

**DOI:** 10.1001/jamanetworkopen.2023.47323

**Published:** 2023-12-12

**Authors:** Yong-Soo Seo, Jin-Sung Yuk

**Affiliations:** 1 Department of Obstetrics and Gynecology, Sanggye Paik Hospital, School of Medicine, Inje University, Seoul, Korea

## Abstract

**IMPORTANCE:**

Prior research about the association between hysterectomy and osteoporosis risk had limitations.

**OBJECTIVE:**

To assess osteoporosis and fracture risk among female patients who underwent hysterectomy due to benign conditions.

**DESIGN, SETTING, AND PARTICIPANTS:**

In this retrospective cohort study, female patients aged 40 to 59 years with benign hysterectomy between 2003 and 2011 were selected from Korean National Health Insurance Data and matched by 1:1 propensity score with female patients who had health checkups and indicated that they had not had a hysterectomy. A Cox proportional hazard model was used to analyze osteoporosis and fracture risk, with participants monitored until December 31, 2020. Data analysis was performed from July 16, 2022, to January 12, 2023.

**EXPOSURES:**

Hysterectomy with or without adnexal surgical procedure.

**MAIN OUTCOMES AND MEASURES:**

The primary outcome was the risk of osteoporosis. Secondary outcomes included the risk of vertebral fracture, hip fracture, other fractures, and total fracture.

**RESULTS:**

The study population included 25 910 patients; the median (IQR) age was 47 (44-50) years, and median (IQR) follow-up period was 10.9 (9.4-12.7) years. In the stratified-extended Cox proportional analysis, female patients who underwent hysterectomy without an adnexal surgical procedure were associated with a higher risk of osteoporosis within 7 years compared with female patients who did not undergo hysterectomy (hazard ratio [HR], 1.28 [95% CI, 1.19-1.37]); the analysis was divided into 7 years due to a violation of the Cox assumption, and the risk did not differ after 7 years (HR, 0.99 [95% CI, 0.93-1.06]). However, the hysterectomy group with an adnexal surgical procedure had an association with higher risk of osteoporosis compared with the nonhysterectomy group both within 7 years of study entry (HR, 1.56 [95% CI, 1.33-1.82]) and after 7 years (HR, 1.20 [95% CI, 1.04-1.40]). In the hysterectomy group without an adnexal surgical procedure, the risks of vertebral fracture, hip fracture, and total fracture were similar to those in the nonhysterectomy group. Similar trends were observed in the hysterectomy group with an adnexal surgical procedure.

**CONCLUSIONS AND RELEVANCE:**

Hysterectomy without an adnexal surgical procedure was associated with an increased osteoporosis risk within 7 years, but not afterwards, compared with the nonhysterectomy group. Hysterectomy was not associated with vertebral and hip fractures.

## Key Points

**Question:**

Are patients who undergo hysterectomy at higher risk of osteoporosis and fractures?

**Findings:**

This cohort study of 25 910 patients found that hysterectomy without an adnexal surgical procedure was associated with an increased risk of osteoporosis within 7 years, but not afterwards, compared with nonhysterectomy. Hysterectomy with or without an adnexal surgical procedure was not associated with any fractures.

**Meaning:**

Hysterectomy without an adnexal surgical procedure was associated with an increased risk of osteoporosis.

## Teaser

This cohort study assesses osteoporosis and fracture risk among female patients in Korea who underwent hysterectomy due to benign conditions.

## Introduction

Osteoporosis is a bone disease that causes bones to become brittle and more likely to break. It involves reduced bone mineral density (BMD), decreased bone mass, microstructural destruction, and poor bone quality.^1^ The prevalence of osteoporosis among women older than 50 years ranges from 15.4% to 16.5% in the United States, which is much higher than the rate among men.^2,3^ Osteoporosis-related fractures are a leading cause of death, disability, and medical costs in older women.^4^ Risk factors for osteoporosis include older age, low body mass index (BMI), smoking, and heavy alcohol consumption.^1,5^ In particular, estrogen deficiency is a substantial risk factor for osteoporosis in women experiencing menopause.^1,6^ Estrogen deficiency leads to enhanced receptor activator of nuclear factor κ-B (RANK) ligand production by osteocytes, which stimulates osteoclast activity and accelerates bone resorption, exceeding the rate of bone formation by osteoblasts.^1,6,7^

Hysterectomy is a common gynecological procedure for uterine fibroids, endometriosis, menstrual abnormalities, and uterine prolapse.^8,9^ In the United States, approximately half of all hysterectomies are performed together with oophorectomy.^10^ Bilateral oophorectomy (BO) is a known risk factor for osteoporosis, as it leads to a substantial decrease in estrogen levels.^10,11^ Additionally, postmenopausal BO negatively affects the conversion of testosterone to estrogen, further contributing to the development of osteoporosis.^12^

There is no consensus on how hysterectomy with ovarian conservation affects ovarian function. Hysterectomy involves the disruption of the utero-ovarian ligament and the division of the uterine artery branch that provides blood to the ovaries, leading to a potential reduction in blood supply to the ovaries.^13,14,15^ However, alternative perspectives suggest that ovarian blood supply may increase after hysterectomy with ovarian preservation, protecting ovarian function due to enhanced ovarian volume and reduced ovarian pulsatility index.^16^

Previous studies have explored the risk of osteoporosis and fractures in female patients who have undergone hysterectomy.^17,18^ However, these studies did not control for menopausal status, BMI, smoking, and alcohol use, which are risk factors for osteoporosis.^17,18^ This study hypothesized that hysterectomy was associated with a higher risk of osteoporosis and fractures.

The primary objective of this study was to determine the risk of osteoporosis following hysterectomy with or without an adnexal surgical procedure. The secondary aim was to assess the fracture risk (hip, vertebral, other, total fractures) after hysterectomy with or without an adnexal surgical.

## Methods

### Ethics

This cohort study received ethical approval from the institutional review board of Sanggye Paik Hospital. Informed consent was not required in compliance with the Bioethics and Safety Act of South Korea. We followed the Strengthening the Reporting of Observational Studies in Epidemiology (STROBE) reporting guideline.

### Database

The National Health Insurance Service (NHIS), which is mandatory for all citizens, provides single-payer health care to most residents in South Korea.^19^ This database contains demographic data, such as the sex and age of the insured person, the health insurance plan, and the codes for their diagnoses, medications, and surgical procedures.^19^ The NHIS offers comprehensive health screening services to South Korean citizens at no cost, providing valuable measurement data and health history information.^19^ We used NHIS data from 2002 to 2020 for this retrospective cohort study.

### Participant Selection

We selected and analyzed the participants based on the *International Statistical Classification of Diseases and Related Health Problems, Tenth Revision (ICD-10)* and the Korea Health Insurance Medical Care Exposes (2012 version, 2020 version). The study included a hysterectomy group (female patients aged 40 to 59 years who had their uterus removed due to benign conditions between 2002 and 2011) and a nonhysterectomy group (female patients aged 40 to 59 years who had a health checkup at the NHIS during the same period). The nonhysterectomy group included only those female patients who indicated in the health checkup questionnaire that they had not had a hysterectomy.

We performed stratified random sampling in 5-year age intervals, selecting 25% to accommodate the NHIS analytics server’s capacity. For washout, we excluded female patients who had undergone either a health checkup or a hysterectomy due to benign conditions in 2002. We also excluded participants who received a diagnosis code for cancer (any Cxx), osteoporosis (M80-M85), or fracture (S02, S12, S22, S32, S42, S52, S62, S72, S82, S92, T02, T08, T10, T12) from a medical institution within 365 days of joining the study.

We matched comparison groups 1:1 by propensity score using variables in Table 1. The study participants were monitored until December 31, 2020.

**Table 1.** Characteristics of Study Participants With and Without Hysterectomy After Propensity Score Matching

Variable	Participants, No. (%)			*P* value	SMD
	Nonhysterectomy (n = 12 955)	Hysterectomy (n = 12 955)	Total (N = 25 910)		
Follow-up period, median (IQR), y	10.9 (9.4-12.6)	10.9 (9.5-12.8)	10.9 (9.4-12.7)	.93	.02
Age, median (IQR), y	48 (44-50)	47 (44-49)	47 (44-50)	.22	.10
Age at inclusion, y					
40-44	3483 (26.9)	3349 (25.9)	6832 (26.4)	<.001	.21
45-49	5261 (40.6)	6400 (49.4)	11 661 (45)		
50-54	3449 (26.6)	2757 (21.3)	6206 (24)		
55-59	762 (5.9)	449 (3.5)	1211 (4.7)		
Year of inclusion					
2003-2005	1046 (8.1)	1467 (11.3)	2513 (9.7)	<.001	.11
2006-2008	4628 (35.7)	4341 (33.5)	8969 (34.6)		
2009-2011	7281 (56.2)	7147 (55.2)	14 428 (55.7)		
BMI, median (IQR)	23.6 (21.7-25.7)	23.5 (21.8-25.7)	23.6 (21.7-25.7)	.08	.01
BMI					
<18.5	215 (1.7)	204 (1.6)	419 (1.6)	.001	.04
18.5-22.9	5129 (39.6)	5229 (40.4)	10 358 (40)		
23-24.9	3241 (25)	3292 (25.4)	6533 (25.2)		
25-29.9	3761 (29)	3713 (28.7)	7474 (28.8)		
≥30	609 (4.7)	517 (4)	1126 (4.3)		
Low SES	46 (0.4)	59 (0.5)	105 (0.4)	.22	.02
Rural area	9157 (70.7)	9252 (71.4)	18 409 (71)	.20	.02
CCI					
1	1978 (15.3)	1885 (14.6)	3863 (14.9)	.02	.03
≥2	466 (3.6)	430 (3.3)	896 (3.5)		
Parity					
1	1722 (13.3)	1648 (12.7)	3370 (13)	<.001	.09
2	8892 (68.6)	8624 (66.6)	17 516 (67.6)		
≥3	755 (5.8)	712 (5.5)	1467 (5.7)		
Age 13 y or older at menarche	10 106 (78)	10 041 (77.5)	20 147 (77.8)	.16	.01
Menopause before inclusion	1877 (14.5)	1676 (12.9)	3553 (13.7)	<.001	.05
Smoking					
Past	205 (1.6)	182 (1.4)	387 (1.5)	.44	.02
Current	522 (4.0)	518 (4.0)	1040 (4.0)		
Alcohol, d/wk^a^					
1-2	3645 (28.1)	3545 (27.4)	7190 (27.7)	<.001	.05
3-6	130 (1.0)	199 (1.5)	329 (1.3)		
Daily	70 (0.5)	65 (0.5)	135 (0.5)		
Physical exercise, d/wk^b^					
1-2	2399 (18.6)	2494 (19.3)	4893 (19)	.11	.03
3-4	1267 (9.8)	1262 (9.8)	2529 (9.8)		
5-6	407 (3.2)	364 (2.8)	771 (3)		
Daily	372 (2.9)	417 (3.2)	789 (3.1)		
Diabetes	1214 (9.4)	1220 (9.4)	2434 (9.4)	.92	<.01
Hypertension	2439 (18.8)	2398 (18.5)	4837 (18.7)	.52	<.01
Dyslipidemia	1996 (15.4)	1889 (14.6)	3885 (15)	.06	.02
MHT before inclusion	199 (1.5)	85 (0.7)	284 (1.1)	<.001	.09
Adnexal surgical procedure before inclusion	194 (1.5)	127 (1.0)	321 (1.2)	<.001	.05
Uterine fibroids	9690 (74.8)	9595 (74.1)	19 285 (74.4)	<.001	.02
Endometriosis	2179 (16.8)	2287 (17.7)	4466 (17.2)	<.001	.02
MHT after inclusion^c^	199 (1.5)	530 (4.1)	729 (2.8)	<.001	.02

Abbreviations: BMI, body mass index (calculated as weight in kilograms divided by height in meters squared); CCI, Charlson comorbidity index; MHT, menopausal hormone therapy; SES, socioeconomic status; SMD, standardized mean difference.

^a^ Number of days per week the participant would have an alcoholic drink.

^b^ Number of days per week the participant would engage in physical exercise.

^c^ This variable was not included in the propensity score matching.

### Outcomes

Osteoporosis was defined as simultaneous BMD test codes (dual-energy x-ray absorptiometry, radiographic absorptiometry, quantitative computed tomography) and osteoporosis diagnosis codes (M80-M82). Female patients who went to clinics more than once for any fracture (vertebral fracture: S12.0-S12.7, S22.0-S22.1, S32.0-S32.2; hip fracture: S72; other fractures: S02, S01.8-S01.9, S22.2-S22.9, S32.3-S32.8, S42, S52, S62, S82, S92, T02, T08, T10, T12) were classified according to each type of fracture (vertebral, hip, other, and total fracture).

### Variables

The study examined factors such as age (grouped in 5-year increments), socioeconomic status (SES) (medical insurance was referred to as medical aid), and self-reported measures of smoking, alcohol drinking, and physical activity levels. We classified participants as living in a rural area if their inclusion area was not urban. BMI was calculated using the criteria of the Asia-Pacific perspective.^20^ We divided age at menarche into less than 13 years and at least 13 years. We also grouped parity into 4 categories: 0, 1, 2, and 3 or more births. Menopausal status was determined through questionnaire responses. Menopausal hormone therapy (MHT) before and after inclusion was defined as the use of such therapy (tibolone, estrogen/progestogen, estrogen) more than 6 months before or after the study participation date. The presence of hypertension (I10-I15), diabetes (E10-E14), hyperlipidemia (E78), uterine fibroids (D25), and endometriosis (N80) was determined based on having visited a medical institution for the respective conditions 2 or more times before study participation. The Charlson comorbidity index (CCI) score was calculated based on diagnosis codes from 1 year before the study participation date to the participation date.^21^ Hysterectomy or adnexal surgical procedure was recorded using surgical procedure codes. Adnexal surgical procedures included the bilateral or unilateral extirpation (cystectomy, oophorectomy, and salpingectomy) of benign adnexal tumors, incision and drainage of ovarian cysts, as well as ovarian wedge resection surgical procedure. Hysterectomy with adnexal surgical procedure was defined as having both procedures on the same day (eTable 1 in Supplement 1). Previous adnexal surgical procedure was defined as having it before the study start date.

### Statistical Analysis

Statistical analysis was performed from July 16, 2022, to January 12, 2023, using R version 3.5.1 (R Foundation for Statistical Computing). Two-tailed *P* < .05 was considered statistically significant. We compared variables with χ^2^, Fisher exact, *t*, and Wilcoxon tests before matching and Cochran-Mantel-Haenszel, paired *t*, and Wilcoxon tests after matching.

We used a stratified Cox regression analysis to estimate the risk of osteoporosis and fracture associated with hysterectomy; and the Schoenfeld residuals test to check the proportional hazards assumption in Cox regression. If the proportional hazards assumption was not met, we performed an extended Cox analysis using the step function. The study participation date was the date of the hysterectomy for the hysterectomy group and the first day of the health checkup for the nonhysterectomy group. The censoring date was the earliest date of the following: the first day that osteoporosis or fracture was identified in the health insurance data, the date of death, or the last day of visiting a health care clinician for any illness. We removed missing values by listwise deletion during propensity score matching. To confirm the robustness of the study results, we performed a stratified Cox regression analysis of female patients with uterine fibroids, endometriosis, and those who had undergone hysterectomy (sensitivity test).

## Results

We included 25% of 4 010 228 female patients with NHIS health checkups or hysterectomy during 2003 to 2011. We matched 12 955 patients with hysterectomy with 12 955 patients without hysterectomy by propensity score (Figure). The median (IQR) age was 47 (44-50) years, and median (IQR) follow-up was 10.9 (9.4-12.7) years. Of the female patients in the hysterectomy group, 10 749 (83.0%) had hysterectomy without an adnexal surgical procedure and 2206 (17.0%) had hysterectomy with an adnexal surgical procedure. After the study entry date, the use of MHT was higher in the hysterectomy group (530 female patients [4.1%]) compared with the nonhysterectomy group (199 female patients [1.5%]). Table 1 and eTable 2 in Supplement 1 show patient characteristics before and after matching.

**Figure.**
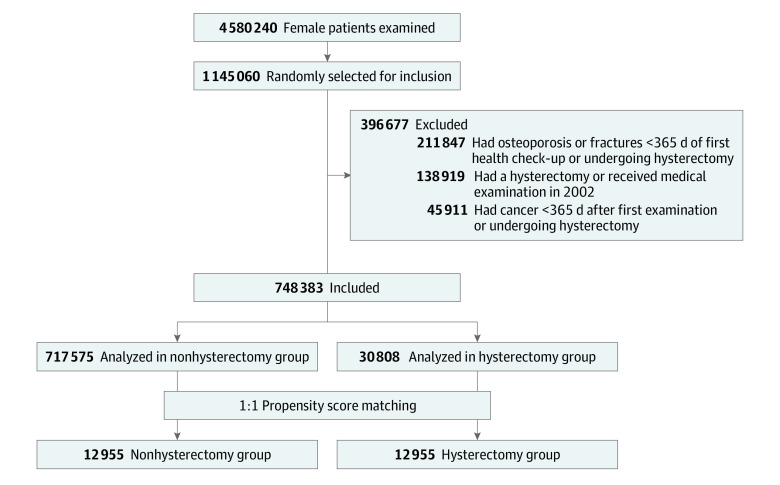
Flowchart of the Risk of Osteoporosis in the Hysterectomy and Nonhysterectomy Groups Using National Health Insurance Data From 2002 to 2020 in South Korea

Osteoporosis was more common in female patients with hysterectomy (2488 [19.2%]) than without (2194 [16.9%]; *P* < .001), but fracture rates were similar (2735 [21.1%] vs 2769 [21.4%]; *P* = .61). See Table 2 for details.

**Table 2.** Incidence of Osteoporosis and Fractures in Hysterectomy and Nonhysterectomy Groups

Variable	Participants, No. (%)			*P* value
	Nonhysterectomy (n = 12 955)	Hysterectomy (n = 12 955)	Total (N = 25 910)	
Vertebral fracture	348 (2.7)	316 (2.4)	664 (2.6)	.22
Hip fracture	17 (0.1)	12 (0.1)	29 (0.1)	.46
Other fracture	2548 (19.7)	2540 (19.6)	5088 (19.6)	.91
Total fracture	2769 (21.4)	2735 (21.1)	5504 (21.2)	.61
Osteoporosis	2194 (16.9)	2488 (19.2)	4682 (18.1)	<.001

The Cox proportional analysis of osteoporosis risk violated the proportional hazards assumption. Therefore, we used Schoenfeld residual plots to confirm that the proportional hazards were satisfied when stratified by 7 years after study entry. Within 7 years, compared with the nonhysterectomy group, osteoporosis risk was higher for hysterectomy with (hazard ratio [HR], 1.56 [95% CI, 1.33-1.82]) or without (HR, 1.28 [95% CI, 1.19-1.37]) an adnexal surgical procedure (Table 3; eFigure in Supplement 1). After 7 years, only hysterectomy with an adnexal surgical procedure was associated with a higher osteoporosis risk (HR, 1.20 [95% CI, 1.04-1.40]), while the osteoporosis risk of hysterectomy without adnexal surgical procedure was similar to nonhysterectomy (HR, 0.99 [95% CI, 0.93-1.06]). Fracture risks were similar in the hysterectomy group, regardless of adnexal surgical procedure, compared with the nonhysterectomy group (Table 3).

**Table 3.** Risk of Osteoporosis and Fractures in the Hysterectomy and Nonhysterectomy Groups Using Cox Proportional Hazard Analysis of National Health Insurance Data From 2002 to 2020 in South Korea

Variable	Unadjusted		Adjusted^a^	
	HR (95% CI)	*P* value	HR (95% CI)^a^	*P* value
Vertebral fracture				
Nonhysterectomy	1 [Reference]	NA	1 [Reference]	NA
Hysterectomy without adnexal surgical procedure	0.91 (0.77-1.09)	.30	1.01 (0.83-1.21)	.96
Hysterectomy with adnexal surgical procedure	0.92 (0.60-1.41)	.71	1.02 (0.65-1.60)	.94
Hip fracture				
Nonhysterectomy	1 [Reference]	NA	1 [Reference]	NA
Hysterectomy without adnexal surgical procedure	0.69 (0.30-1.62)	.40	1.48 (0.28-7.78)	.65
Hysterectomy with adnexal surgical procedure	0.50 (0.09-2.73)	.42	0.43 (0.01-13.82)	.64
Other fracture				
Nonhysterectomy	1 [Reference]	NA	1 [Reference]	NA
Hysterectomy without adnexal surgical procedure	0.95 (0.89-1.02)	.16	0.96 (0.90-1.03)	.27
Hysterectomy with adnexal surgical procedure	0.98 (0.85-1.14)	.80	1.0 (0.86-1.17)	.97
Total fracture				
Nonhysterectomy	1 [Reference]	NA	1 [Reference]	NA
Hysterectomy without adnexal surgical procedure	0.95 (0.89-1.01)	.09	0.96 (0.90-1.02)	.21
Hysterectomy with adnexal surgical procedure	0.96 (0.83-1.1)	.52	0.98 (0.85-1.14)	.84
Osteoporosis				
<7 y				
Nonhysterectomy	1 [Reference]	NA	1 [Reference]	NA
Hysterectomy without adnexal surgical procedure	1.28 (1.19-1.37)	<.001	1.33 (1.23-1.43)	<.001
Hysterectomy with adnexal surgical procedure	1.56 (1.33-1.82)	<.001	1.56 (1.32-1.84)	<.001
≥7 y				
Nonhysterectomy	1 [Reference]	NA	1 [Reference]	NA
Hysterectomy without adnexal surgical procedure	0.99 (0.93-1.06)	.81	1.05 (0.98-1.13)	.18
Hysterectomy with adnexal surgical procedure	1.21 (1.04-1.40)	.02	1.19 (1.02-1.40)	.03

Abbreviations: HR, hazard ratio; NA, not applicable.

^a^ This stratified-extended Cox proportional analysis adjusted for age, year at inclusion, body mass index, socioeconomic status, region, Charlson comorbidity index, parity, age at menarche, menopause before inclusion, smoking, alcohol, physical exercise, diabetes, hypertension, dyslipidemia, menopausal hormone therapy before inclusion, adnexal surgical procedure before inclusion, uterine fibroids, and endometriosis.

The age-specific analyses found that female patients aged 40 to 49 years who had a hysterectomy without adnexal surgical procedure were associated with higher risk of osteoporosis within 7 years of study entry (HR, 1.42 [95% CI, 1.29-1.58]) (Table 4). Female patients aged 50 to 59 years who underwent hysterectomy without an adnexal surgical procedure were also associated with a significantly increased risk of osteoporosis (HR, 1.17 [95% CI, 1.01-1.34]). However, in both age groups (female patients aged 40 to 49 years and 50 to 59 years), the overall fracture risk in the hysterectomy group with or without an adnexal surgical procedure was comparable with that of the nonhysterectomy group.

**Table 4.** Risk of Osteoporosis and Fractures in the Hysterectomy and Nonhysterectomy Groups by Age Using National Health Insurance Data From 2002 to 2020 in South Korea

Variable	Aged 40-49 y				Aged 50-59 y			
	Unadjusted		Adjusted^a^		Unadjusted		Adjusted^a^	
	HR (95% CI)	*P* value	HR (95% CI)	*P* value	HR (95% CI)	*P* value	HR (95% CI)	*P* value
Vertebral fracture								
Nonhysterectomy	1 [Reference]	NA	1 [Reference]	NA	1 [Reference]	NA	NA	NA
Hysterectomy without adnexal surgical procedure	0.99 (0.78-1.25)	.91	0.99 (0.77-1.27)	.92	0.96 (0.69-1.33)	.80	NA	NA
Hysterectomy with adnexal surgical procedure	1.11 (0.59-2.1)	.75	1.06 (0.53-2.12)	.86	0.84 (0.43-1.64)	.61	NA	NA
Hip fracture								
Nonhysterectomy	1 [Reference]	NA	1 [Reference]	NA	1 [Reference]	NA	NA	NA
Hysterectomy without adnexal surgical procedure	2.50 (0.49-12.89)	.27	1.22 (0.16-9.56)	.85	0.50 (0.13-2.00)	.33	NA	NA
Hysterectomy with adnexal surgery	NA	NA	NA	NA	1.00 (0.14-7.10)	>.99	NA	NA
Other fracture								
Nonhysterectomy	1 [Reference]	NA	1 [Reference]	NA	1 [Reference]	NA	1 [Reference]	NA
Hysterectomy without adnexal surgical procedure	0.96 (0.89-1.05)	41	0.95 (0.87-1.03)	.21	1.06 (0.92-1.21)	.45	1.06 (0.91-1.22)	.47
Hysterectomy with adnexal surgical procedure	0.97 (0.79-1.19)	.79	0.94 (0.76-1.17)	.59	1.08 (0.83-1.42)	.57	1.08 (0.82-1.43)	.59
Total fracture								
Nonhysterectomy	1 [Reference]	NA	1 [Reference]	NA	1 [Reference]	NA	1 [Reference]	NA
Hysterectomy without adnexal surgical procedure	0.97 (0.89-1.05)	.44	0.95 (0.88-1.04)	.26	1.03 (0.90-1.18)	.68	1.04 (0.90-1.2)	.58
Hysterectomy with adnexal surgical procedure	0.99 (0.81-1.21)	.91	0.97 (0.79-1.19)	.75	1.00 (0.77-1.3)	.98	1.04 (0.80-1.4)	.76
Osteoporosis								
Nonhysterectomy	1 [Reference]	NA	1 [Reference]	NA	1 [Reference]	NA	1 [Reference]	NA
Hysterectomy without adnexal surgical procedure	NA	NA	NA	NA	1.17 (1.01-1.34)	.03	1.22 (1.06-1.42)	.01
<7 y	1.42 (1.89-1.58)	<.001	1.38 (1.24-1.53)	<.001	NA	NA	NA	NA
≥7 y	1.02 (0.94-1.12)	.62	0.99 (0.90-1.08)	.76	NA	NA	NA	NA
Hysterectomy with adnexal surgical procedure	NA	NA	NA	NA	1.39 (1.07-1.81)	.01	1.36 (1.03-1.77)	.03
<7 y	1.68 (1.32-2.13)	<.001	1.52 (1.18-1.96)	<.001	NA	NA	NA	NA
≥7 y	1.27 (1.02-1.58)	.03	1.25 (1.00-1.56)	.05	NA	NA	NA	NA

Abbreviations: HR, hazard ratio; NA, not applicable.

^a^ This stratified-extended Cox proportional analysis adjusted for age, year at inclusion, body mass index, socioeconomic status, region, Charlson comorbidity index, parity, age at menarche, menopause before inclusion, smoking, alcohol, physical exercise, diabetes, hypertension, dyslipidemia, menopausal hormone therapy before inclusion, adnexal surgical procedure before inclusion, uterine fibroids, and endometriosis.

Osteoporosis incidence was 177 per 10 000 person-years in the hysterectomy group and 157 per 10 000 person-years in the nonhysterectomy group (eTable 3 in Supplement 1). In a sensitivity test that included only (1) female patients in the nonhysterectomy group with uterine fibroids or endometriosis and (2) female patients in the hysterectomy group, hysterectomy without an adnexal surgical procedure was associated with a higher risk of osteoporosis (HR, 1.30 [95% CI, 1.20-1.41]) in the first 7 years but not after (HR, 0.97 [95% CI, 0.9-1.05]) (eTable 4 in Supplement 1). Hysterectomy with an adnexal surgical procedure was associated with osteoporosis regardless of duration.

## Discussion

This study found that hysterectomy with or without an adnexal surgical procedure was associated with a higher risk of osteoporosis within 7 years of the procedure. However, after 7 years, the risk of osteoporosis in the hysterectomy group without an adnexal surgical procedure was similar to that in the nonhysterectomy group. Notably, hysterectomy with an adnexal surgical procedure was associated with a slightly elevated risk of osteoporosis 7 years post surgery, albeit lower than the risk within the initial 7-year time frame. Furthermore, the hysterectomy groups with or without an adnexal surgical procedure did not differ from the nonhysterectomy group regarding the risk of vertebral and hip fractures.

### Hysterectomy and Ovarian Function

Both the ovarian and uterine arteries supply blood to the ovaries.^22^ Hysterectomy with ovarian conservation may compromise ovarian function by decreasing ovarian blood supply or eliminating the uterine paracrine effect. The first hypothesis is that the uterine artery branch to the ovaries and the utero-ovarian ligament is severed by hysterectomy, affecting ovarian function.^13^ The second hypothesis posits that the uterus plays a paracrine role in safeguarding against follicle depletion, as supported by the elevated follicle-stimulating hormone levels observed in individuals who undergo endometrial ablation.^23^ Impaired ovarian function may lead to estrogen deficiency, which stimulates the production of RANK ligands that activate osteoclasts. The increasing number of RANK ligands result in bone resorption, exceeding bone formation by osteoblasts, causing a rapid phase of bone loss and bone fracture.^6,7,24^

The impact of hysterectomy with ovarian preservation on ovarian function is unclear. Some studies have shown that ovarian function is impaired after hysterectomy with ovarian preservation. Moorman et al^14^ reported an increased risk of ovarian failure (HR, 1.74 [95% CI, 1.14-2.65]) in a 5-year follow-up study. Trabuco et al^15^ detected a decrease in the ovarian reserve by assessing anti-müllerian hormone (AMH) levels 1 year after hysterectomy with ovarian preservation. However, other studies found that ovarian function was not impaired after hysterectomy with ovarian preservation. Abdelazim et al^16^ reported no difference in AMH levels before or 1 year after hysterectomy with ovarian conservation. Lee et al^25^ reported no difference in AMH levels assessed 3 months after hysterectomy. These studies, however, had limitations, such as a small sample size (n = 32) or the lack of a control group.^16,25^

### Hysterectomy and Osteoporosis and/or Fracture

A few studies have reported that hysterectomy with ovarian preservation may cause osteoporosis, even though its effects on ovarian function are controversial. For example, Choi et al^17^ found that this type of hysterectomy increased the risk of osteoporosis (HR, 1.45 [95% CI, 1.37-1.53]). Similarly, a Taiwanese population-based observational study reported that hysterectomy with ovarian preservation increased the risk of osteoporosis (HR, 1.52 [95% CI, 1.36-1.71]) and vertebral fracture (HR, 4.92 [95% CI, 3.78-6.40]).^18^ However, this study also noted that the association of hysterectomy without an adnexal surgical procedure with osteoporosis was only significant within 7 years of the procedure and did not affect the risk of vertebral and hip fractures in the hysterectomy group compared with the nonhysterectomy group.

The difference in results between previous studies and our study may be attributed to 2 main factors. First, the disparity in follow-up times played a substantial role. Previous studies had a mean follow-up period of 5.25 years and a median follow-up of 6.66 years for the hysterectomy group.^17,18^ In contrast, our study had a much more extended median (IQR) follow-up period of 10.9 (9.4-12.6) years, approximately twice as long as that in the previous studies. This extended follow-up duration enabled our study to capture changes in osteoporosis and fracture risk, specifically at the 7-year mark post hysterectomy. The second reason for the discrepancy in findings is likely due to differences in methods. Previous studies did not adequately account for various risk factors for osteoporosis, such as BMI, smoking, alcohol consumption, physical exercise level, and menopause.^1,5,17,18^ In contrast, our study considered these as confounding factors and adjusted for them in the analysis. Additionally, the study by Choi et al^17^ had additional methodological flaws.^26^ First, their study included female patients who underwent hysterectomy due to malignant indications, some of whom were diagnosed with ovarian or uterine cancer. Since weight loss caused by chemotherapy is a risk factor for osteoporosis and can affect the progression of osteoporosis, it is unreasonable to directly compare the hysterectomy group with cancer to the control group without cancer.^6^ These limitations should be acknowledged in the interpretation of the results.

This study has strengths. First, propensity score matching was used to mitigate bias associated with multiple influential factors, including age, region, SES, CCI score, parity, BMI, smoking status, alcohol consumption, physical exercise level, adnexal surgical procedure, and MHT. Previous studies did not consider some of these important variables despite their known influence on osteoporosis risk.^17,18,27^ Second, in contrast to previous research relying solely on diagnostic codes, this study adopted a comprehensive approach by combining diagnostic codes with BMD testing to accurately define osteoporosis. This integrated approach aimed to minimize potential coding errors associated with diagnostic codes alone.

### Time-Dependent Association

Our study had a unique feature regarding the association of hysterectomy with osteoporosis risk, especially at the 7-year mark. Within 7 years following hysterectomy without an adnexal surgical procedure, there was an observed increase in the risk of osteoporosis. However, beyond 7 years, the difference in osteoporosis risk was not significant. This finding suggests that some factors may mitigate the negative association of hysterectomy without an adnexal surgical procedure with osteoporosis over time. It is important to note that such a time-dependent association cannot be reasonably attributed to the hysterectomy procedure alone. One possible factor is the use of posthysterectomy treatments, such as MHT, calcium supplementation, and/or vitamin D supplementation. Some female patients who undergo a hysterectomy may experience decreased ovarian function and menopausal symptoms, such as night sweats, hot flashes, and vaginal dryness. MHT may be administered to alleviate these symptoms.^14,15,28^ MHT effectively prevents osteoporosis and fractures, which could explain the decrease in osteoporosis risk over time.^28^ Additionally, the combined effects of hysterectomy itself, MHT use after hysterectomy, and osteoporosis after hysterectomy probably contribute to fracture risk. In this study, hysterectomy was not associated with fracture risk, possibly because posthysterectomy osteoporosis treatments reduced the risk. In our study, MHT use was higher in the hysterectomy group (4.1%) than in the nonhysterectomy group (1.5%) after the study entry date. Further investigations should consider the variables affecting bone health after hysterectomy, including MHT, calcium, and vitamin D preparations.

### Analysis by Age

Younger female patients who had hysterectomy with or without an adnexal surgical procedure had a higher risk of osteoporosis within 7 years of study entry than female patients aged 50 to 59 years. This result agrees with previous research, such as the Choi et al^17^ study, which indicated a higher osteoporosis risk in female patients aged 40 to 44 years who had hysterectomy with or without BO compared with older female patients (HR, 1.84 [95% CI, 1.61-2.1]).^17^ Likewise, Yeh et al^18^ found that female patients aged in their 30s and 40s had a higher risk of hysterectomy with or without oophorectomy than female patients aged in their 50s and 60s.^18^ The mean age of menopause for Korean women is 50.4 years.^29^ Oophorectomy before 45 years of age is a documented risk factor for osteoporosis.^11^ Thus, hysterectomy with or without an adnexal surgical procedure appears to be associated with osteoporosis risk at a relatively young age.^30,31^

### Limitations

Certain limitations exist within this study. First, due to available health insurance data constraints, it was unfeasible to determine the specific surgical technique used for an adnexal surgical procedure. Although this study encompassed procedures such as BO, unilateral ovarian cystectomy, and unilateral salpingectomy, distinguishing among these procedures was not feasible. This limitation is consistent with a similar study conducted by Choi et al.^17^ Second, this study did not account for crucial factors, including thyroid disease, rheumatoid disease, Crohn disease, family history of osteoporosis, steroid usage, calcium intake, and vitamin D supplementation. These variables necessitate further investigation to comprehensively address their potential effect on osteoporosis risk.

## Conclusions

This retrospective cohort study found that among female patients aged 40 to 50 years, the hysterectomy group without an adnexal surgical procedure was associated with a higher osteoporosis risk within the first 7 years after the procedure compared with the nonhysterectomy group. However, this risk did not differ significantly after 7 years. Hysterectomy with an adnexal surgical procedure was also associated with an increased osteoporosis risk; however, hysterectomy, either with or without an adnexal surgical procedure, did not show an association with an increased risk of vertebral or hip fractures. Therefore, patients should be counseled on this potential association.

## Notes

Supplement 1. **eTable 1.** Diagnosis and Procedure Codes Used in the Study **eTable 2.** Comparison of Characteristics Between Participants With and Without Hysterectomy in the Study (Before Propensity Score Matching) **eTable 3.** Osteoporosis Case/Person-Years in Hysterectomy and Nonhysterectomy Groups Using National Health Insurance Data From 2002 to 2020 in South Korea **eTable 4.** Risk of Osteoporosis and Fracture in the Nonhysterectomy With Uterine Fibroids or Endometriosis and Hysterectomy Groups Using Cox Proportional Hazards Analysis (Sensitivity Test) **eFigure.** Kaplan-Meier Plot of Incidence of Osteoporosis in Hysterectomy and Nonhysterectomy Groups Using National Health Insurance Data From 2002 to 2020 in South Korea. (Stratified Log-Rank Test: P-value <.001)

Supplement 2.
Data Sharing Statement
