# Supplementary material for: Osteoporosis and Fracture Risk Following Benign Hysterectomy Among Female Patients in Korea
Source: JAMA Netw Open. 2023 Dec 12;6(12):e2347323. doi: 10.1001/jamanetworkopen.2023.47323 (PMC10716721; doi:10.1001/jamanetworkopen.2023.47323)
Supplement: Supplement 1. — eTable 1. Diagnosis and Procedure Codes Used in the Study eTable 2. Comparison of Characteristics Between Participants With and Without Hysterectomy in the Study (Before Propensity Score Matching) eTable 3. Osteoporosis Case/Person-Years in Hysterectomy and Nonhysterectomy Groups Using National Health Insurance Data From 2002 to 2020 in South Korea eTable 4. Risk of Osteoporosis and Fracture in the Nonhysterectomy With Uterine Fibroids or Endometriosis and Hysterectomy Groups Using Cox Proportional Hazards Analysis (Sensitivity Test) eFigure. Kaplan-Meier Plot of Incidence of Osteoporosis in Hysterectomy and Nonhysterectomy Groups Using National Health Insurance Data From 2002 to 2020 in South Korea. (Stratified Log-Rank Test: P-value <.001) [file jamanetwopen-e2347323-s001.pdf]

Supplemental Online Content

Seo Y, Yuk J. Osteoporosis and fracture risk following benign hysterectomy among female patients in Korea. *JAMA Netw Open.* 2023;6(12):e2347323. doi:10.1001/jamanetworkopen.2023.47323

eTable 1. Diagnosis and Procedure Codes Used in the Study

eTable 2. Comparison of Characteristics Between Participants With and Without Hysterectomy in the Study (Before Propensity Score Matching)

eTable 3. Osteoporosis Case/Person-Years in Hysterectomy and Nonhysterectomy Groups Using National Health Insurance Data From 2002 to 2020 in South Korea

eTable 4. Risk of Osteoporosis and Fracture in the Nonhysterectomy With Uterine Fibroids or Endometriosis and Hysterectomy Groups Using Cox Proportional Hazards Analysis (Sensitivity Test)

eFigure. Kaplan-Meier Plot of Incidence of Osteoporosis in Hysterectomy and Nonhysterectomy Groups Using National Health Insurance Data From 2002 to 2020 in South Korea. (Stratified Log-Rank Test: P-value <.001)

This supplemental material has been provided by the authors to give readers additional information about their work.

© 2023 Seo Y et al. *JAMA Network Open.*

eTable 1. Diagnosis and procedure codes used in the study

Diagnosis name	Diagnosis code
Osteoporosis (for outcome)	M80~M82
Osteoporosis (for excluding)	M80~M85
Vertebral fracture (for outcome)	S12.0~S12.7/S22.0~S22.1/S32.0~S32.2
Hip fracture (for outcome)	S72
Other fracture (for outcome)	S02/S01.8~S01.9/S22.2~S22.9/S32.3~S32.8/S42/S52/S62/S82/S92/T02/T08/T10/T12
Fracture (for excluding)	S02/S12/S22/S32/S42/S52/S62/S72/S82/S92/T02/T08/T10/T12
Cancers (for excluding)	Cxx

BMD test	Procedure code
Dual-energy X-ray absorptiometry	HC341, HC342
Radiographic absorptiometry	HC345
Quantitative computed tomography	HC343
Peripheral quantitative computed tomography	HC346
Other methods	HC344

Surgery name	Surgery code
Adnexal surgery	R4331: Unilateral adnexectomy
	R4332: Bilateral adnexectomy
	R4421: Extirpation of benign adnexal tumor (including oophorectomy, cystectomy, and salpingectomy)
	R4430: Ovarian wedge resection
	R4435: Incision and drainage of ovarian cyst
Hysterectomy	R4145: Simple hysterectomy without lymphadenectomy

© 2023 Seo Y et al. *JAMA Network Open.*

	R4146: Complex hysterectomy without lymphadenectomy
	R4130: Subtotal hysterectomy

BMD, bone mineral density

© 2023 Seo Y et al. *JAMA Network Open.*

eTable 2. Comparison of characteristics between participants with and without hysterectomy in the study (before propensity score matching)

	Subjects, No. (%)			P-value	SMD	Missing, %
	Nonhysterectomy	Hysterectomy	Total			
Number of women	717,575	30,808	748,383			
Follow-up period, median (IQR), y	11.9 [10-14]	12.3 [10-14.9]	11.9 [10-14]	<.001	.11	0
Age, median (IQR), y	47 [42-51]	46 [44-49]	46 [42-51]	.01	.08	0
Age at inclusion, y				<.001	.56	0
40~44	306,792 (42.8)	10,288 (33.4)	317,080 (42.4)			
45~49	168,598 (23.5)	14,233 (46.2)	182,831 (24.4)			
50~55	162,181 (22.6)	5,520 (17.9)	167,701 (22.4)			
55~60	80,004 (11.1)	767 (2.5)	80,771 (10.8)			
Year at inclusion				<.001	.24	0
2003~2005	172,448 (24)	10,716 (34.8)	183,164 (24.5)			
2006~2008	284,478 (39.6)	10,182 (33)	294,660 (39.4)			
2009~2011	260,649 (36.3)	9,910 (32.2)	270,559 (36.2)			
Median BMI (kg/m^2)	23.1 [21.4-25.3]	23.7 [21.8-25.9]	23.2 [21.4-25.3]	<.001	.11	0
BMI (kg/m2)				<.001	.17	0
<18.5	18,113 (2.5)	471 (1.5)	18,584 (2.5)			
18.5-22.9	320,751 (44.7)	11,835 (38.4)	332,586 (44.4)			
23-24.9	173,871 (24.2)	7,828 (25.4)	181,699 (24.3)			
25-29.9	178,888 (24.9)	9,215 (29.9)	188,103 (25.1)			
≥30	25,952 (3.6)	1,459 (4.7)	27,411 (3.7)			
SES				<.001	.05	0

© 2023 Seo Y et al. *JAMA Network Open.*

Mid~high SES	714,703 (99.6)	30,569 (99.2)	745,272 (99.6)			
Medical aid	2,872 (0.4)	239 (0.8)	3,111 (0.4)			
Region				.14	.01	0
Urban area	209,926 (29.3)	9,134 (29.6)	219,060 (29.3)			
Rural area	507,649 (70.7)	21,674 (70.4)	529,323 (70.7)			
CCI				<.001	.08	0
0	606,417 (84.5)	25,332 (82.2)	631,749 (84.4)			
1	93,088 (13)	4,301 (14)	97,389 (13)			
≥2	18,070 (2.5)	1,175 (3.8)	19,245 (2.6)			
Parity				<.001	.25	0
0	159,241 (22.2)	4,455 (14.5)	163,696 (21.9)			
1	74,253 (10.3)	3,970 (12.9)	78,223 (10.5)			
2	426,580 (59.4)	20,750 (67.4)	447,330 (59.8)			
≥3	57,501 (8)	1,633 (5.3)	59,134 (7.9)			
Age at menarche (years)				<.001	.1	2
<13	180,435 (25.7)	6,482 (21.3)	186,917 (25.5)			
≥13	521,510 (74.3)	23,967 (78.7)	545,477 (74.5)			
Menopause before inclusion				<.001	.09	0
Absent	534,847 (74.5)	24,125 (78.3)	558,972 (74.7)			
Present	182,728 (25.5)	6,683 (21.7)	189,411 (25.3)			
Smoking				.31	.01	10
Never	627,076 (94.5)	12,591 (94.6)	639,667 (94.5)			
Past	10,479 (1.6)	188 (1.4)	10,667 (1.6)			
Current	25,946 (3.9)	525 (3.9)	26,471 (3.9)			
Alcohol (number per week)				<.001	.09	9
None	495,027 (74.1)	9,440 (70.4)	504,467 (74.1)			

© 2023 Seo Y et al. *JAMA Network Open.*

~2/week	162,308 (24.3)	3,686 (27.5)	165,994 (24.4)			
3~6/week	7,117 (1.1)	214 (1.6)	7,331 (1.1)			
Daily	3,282 (0.5)	76 (0.6)	3,358 (0.5)			
Physical exercise (number per week)				<.001	.04	9
None	432,974 (65)	8,652 (64.6)	441,626 (65)			
1~2/week	125,391 (18.8)	2,610 (19.5)	128,001 (18.8)			
3~4/week	62,682 (9.4)	1,314 (9.8)	63,996 (9.4)			
5~6/week	19,400 (2.9)	383 (2.9)	19,783 (2.9)			
Daily	25,902 (3.9)	434 (3.2)	26,336 (3.9)			
DM before inclusion				<.001	.02	0
Absent	663,073 (92.4)	28,319 (91.9)	691,392 (92.4)			
Present	54,502 (7.6)	2,489 (8.1)	56,991 (7.6)			
Hypertension before inclusion				<.001	.05	0
Absent	614,283 (85.6)	25,781 (83.7)	640,064 (85.5)			
Present	103,292 (14.4)	5,027 (16.3)	108,319 (14.5)			
Dyslipidemia before inclusion				<.001	.02	0
Absent	642,179 (89.5)	27,391 (88.9)	669,570 (89.5)			
Present	75,396 (10.5)	3,417 (11.1)	78,813 (10.5)			
MHT before inclusion				<.001	.12	0
Absent	703,244 (98)	30,621 (99.4)	733,865 (98.1)			
Present	14,331 (2)	187 (0.6)	14,518 (1.9)			
Adnexal surgery before inclusion				.95	<.001	0
Absent	711,810 (99.2)	30,562 (99.2)	742,372 (99.2)			
Present	5,765 (0.8)	246 (0.8)	6,011 (0.8)			
Uterine fibroids before inclusion				<.001	1.79	0

© 2023 Seo Y et al. *JAMA Network Open.*

Absent	678,134 (94.5)	9,177 (29.8)	687,311 (91.8)			
Present	39,441 (5.5)	21,631 (70.2)	61,072 (8.2)			
Endometriosis before inclusion				<.001	.54	0
Absent	707,648 (98.6)	25,838 (83.9)	733,486 (98)			
Present	9,927 (1.4)	4,970 (16.1)	14,897 (2)			
MHT after inclusion				<.001	.2	0
Absent	707,052 (98.5)	29,270 (95)	736,322 (98.4)			
Present	10,523 (1.5)	1,538 (5)	12,061 (1.6)			

BMI, body mass index; DM, diabetes mellitus; CCI, Charlson comorbidity index; MHT, menopausal hormone therapy; SES, socioeconomic status, SMD, Standardized mean difference
The data is presented as the number and percentage, or as the median with the 25th and 75th percentiles.

© 2023 Seo Y et al. *JAMA Network Open.*

eTable 3. Osteoporosis case/person-years in hysterectomy and nonhysterectomy groups using National Health Insurance Data from 2002 to 2020 in South Korea

	Nonhysterectomy	Hysterectomy
Total	2,194/140,105 (157)	2,488/140,803 (177)
Age at inclusion (years)		
40~44	324/38,301 (85)	434/38,128 (114)
45~49	857/57,292 (150)	1,219/69,670 (175)
50~54	747/36,589 (204)	679/28,483 (238)
55~59	266/7,923 (336)	156/4,522 (345)
Year at inclusion		
2003~2005	350/14,358 (244)	472/20,417 (231)
2006~2008	971/55,661 (174)	980/51,549 (190)
2009~2011	873/70,086 (125)	1,036/68,837 (151)
BMI (kg/m2)		
<18.5	45/2,268 (198)	64/2,087 (307)
18.5-22.9	946/54,789 (173)	1,081/56,509 (191)
23-24.9	540/35,315 (153)	629/35,886 (175)
25-29.9	578/41,116 (141)	650/40,683 (160)
≥30	85/6,618 (128)	64/5,638 (114)
SES		
Mid~high SES	2,192/139,653 (157)	2,474/140,167 (177)
Low SES	2/452 (44)	14/637 (220)
Region		
Urban area	588/41,197 (143)	697/40,572 (172)
Rural area	1,606/98,907 (162)	1,791/100,232 (179)

© 2023 Seo Y et al. *JAMA Network Open.*

CCI		
0	1,740/113,758 (153)	1,986/116,154 (171)
1	363/21,395 (170)	401/19,976 (201)
≥2	91/4,951 (184)	101/4,673 (216)
Parity		
0 or not respond	389/20,055 (194)	507/24,543 (207)
1	255/17,966 (142)	260/17,311 (150)
2	1,417/94,045 (151)	1,542/90,972 (170)
≥3	133/8,038 (165)	179/7,977 (224)
Age at menarche (years)		
<13	489/31,661 (154)	538/32,851 (164)
≥13	1,705/108,444 (157)	1,950/107,952 (181)
Menopause before inclusion		
Absent	1,715/119,271 (144)	1,999/121,595 (164)
Present	479/20,834 (230)	489/19,209 (255)
Smoking		
Never	2,079/132,485 (157)	2,359/133,308 (177)
Past	27/2,153 (125)	29/1,949 (149)
Current	88/5,467 (161)	100/5,547 (180)
Alcohol (per week)		
None	1,596/98,461 (162)	1,808/99,509 (182)
~2/week	546/39,135 (140)	625/38,150 (164)
3~6/week	32/1,625 (197)	37/2,417 (153)
Daily	20/883 (227)	18/727 (248)
Physical exercise (per week)		
None	1,339/89,474 (150)	1,565/89,952 (174)

© 2023 Seo Y et al. *JAMA Network Open.*

1~2/week	440/26,652 (165)	483/27,341 (177)
3~4/week	240/14,438 (166)	269/14,009 (192)
5~6/week	78/4,478 (174)	66/3,932 (168)
Daily	88/4,413 (199)	93/4,810 (193)
Diabetes before inclusion		
Absent	1,958/127,196 (154)	2,254/127,880 (176)
Present	236/12,908 (183)	234/12,924 (181)
Hypertension before inclusion		
Absent	1,719/113,961 (151)	2,029/115,486 (176)
Present	475/26,143 (182)	459/25,318 (181)
Dyslipidemia before inclusion		
Absent	1,834/119,207 (154)	2,133/121,170 (176)
Present	360/20,898 (172)	355/19,634 (181)
MHT before inclusion		
Absent	2,148/137,979 (156)	2,460/139,978 (176)
Present	46/2,125 (216)	28/825 (339)
Adnexal surgery before inclusion		
Absent	2,173/138,114 (157)	2,471/139,493 (177)
Present	21/1,991 (105)	17/1,310 (130)
Uterine fibroids before inclusion		
Absent	548/35,795 (153)	661/37,362 (177)
Present	1,646/104,310 (158)	1,827/103,442 (177)
Endometriosis before inclusion		
Absent	1,859/117,265 (159)	2,106/116,581 (181)
Present	335/22,839 (147)	382/24,223 (158)
MHT after inclusion[a]		

© 2023 Seo Y et al. *JAMA Network Open.*

Absent	1,995/138,308 (144)	1,958/136,433 (144)
Present	199/1,797 (1,107)	530/4,370 (1,213)

BMI, body mass index; DM, diabetes mellitus; CCI, Charlson comorbidity index; MHT, menopausal hormone therapy; SES, socioeconomic status

The data is presented as the number of cases per 10,000 person-years.

[a] This variable was not included in the propensity score matching.

© 2023 Seo Y et al. *JAMA Network Open.*

eTable 4. Risk of osteoporosis and fracture in the nonhysterectomy with uterine fibroids or endometriosis and hysterectomy groups using Cox proportional hazards analysis (Sensitivity test)

Osteoporosis/Fracture	Unadjusted		Adjusted	
	HR (95% CI)	P-value	HR (95% CI) [a]	P-value
Vertebral fracture				
Nonhysterectomy	1		1	
Hysterectomy without adnexal surgery	0.91 (0.75-1.11)	.34	1.04 (0.83-1.29)	.75
Hysterectomy with adnexal surgery	0.86 (0.54-1.39)	.55	0.91 (0.54-1.52)	.71
Hip fracture				
Nonhysterectomy	1		1	
Hysterectomy without adnexal surgery	0.4 (0.13-1.28)	.12	0.33 (0.06-1.92)	.22
Hysterectomy with adnexal surgery	1 (0.14-7.1)	1	-	-
Other fracture				
Nonhysterectomy	1		1	
Hysterectomy without adnexal surgery	0.93 (0.86-1)	.06	0.94 (0.87-1.01)	.1
Hysterectomy with adnexal surgery	1.01 (0.86-1.19)	.92	1.04 (0.88-1.22)	.67
Total fracture				
Nonhysterectomy	1		1	
Hysterectomy without adnexal surgery	0.93 (0.86-1)	.04	0.94 (0.87-1.01)	.09
Hysterectomy with adnexal surgery	0.98 (0.84-1.15)	.82	1.02 (0.86-1.19)	.85
Osteoporosis				
< 7 years				
Nonhysterectomy	1		1	
Hysterectomy without adnexal surgery	1.3 (1.2-1.41)	<.001	1.35 (1.23-1.47)	<.001

© 2023 Seo Y et al. *JAMA Network Open.*

Hysterectomy with adnexal surgery	1.62 (1.36-1.93)	<.001	1.62 (1.35-1.95)	<.001
≥ 7 years				
Nonhysterectomy	1		1	
Hysterectomy without adnexal surgery	0.97 (0.9-1.05)	.5	1.01 (0.93-1.1)	.81
Hysterectomy with adnexal surgery	1.28 (1.08-1.53)	.01	1.26 (1.04-1.51)	.02

BMI, body mass index; CCI, Charlson comorbidity index; CI, confidence interval; HR, hazard ratio; MHT, menopausal hormone therapy; SES, socioeconomic status

[a] This stratified-extended Cox proportional analysis adjusted for Age, Year at inclusion, BMI, SES, Region, CCI, Parity, Age at menarche, Menopause before inclusion, Smoking, Alcohol, Physical exercise, Diabetes, Hypertension, Dyslipidemia, MHT before inclusion, Adnexal surgery before inclusion, Uterine fibroids, and Endometriosis.

© 2023 Seo Y et al. *JAMA Network Open.*

eFigure 1. Kaplan-Meier plot of incidence of osteoporosis in hysterectomy and nonhysterectomy groups using National Health Insurance Data from 2002 to 2020 in South Korea. (Stratified log-rank test: p-value <.001)

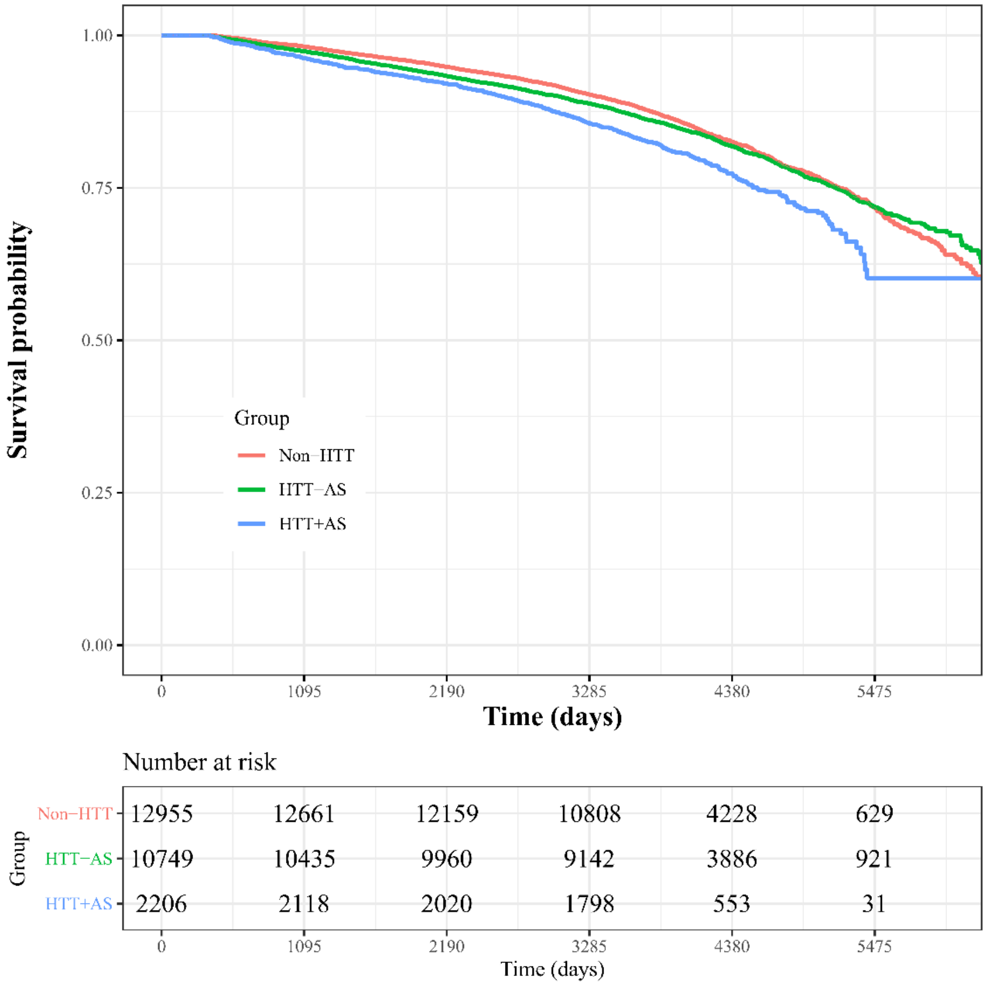

AS, adnexal surgery; HTT, hysterectomy

© 2023 Seo Y et al. *JAMA Network Open.*
