# Supplementary material for: Osteoporosis and Fracture Risk Following Benign Hysterectomy Among Female Patients in Korea
Source: JAMA Netw Open. 2023 Dec 12;6(12):e2347323. doi: 10.1001/jamanetworkopen.2023.47323 (PMC10716721; doi:10.1001/jamanetworkopen.2023.47323)
Supplement: Supplement 2. — Data Sharing Statement [file jamanetwopen-e2347323-s002.pdf]

Data Sharing Statement

Seo. Osteoporosis and Fracture Risk Following Benign Hysterectomy Among Female Patients in Korea. *JAMA Netw Open*. Published December 12, 2023.
doi:10.1001/jamanetworkopen.2023.47323

Data
Data available: No

Additional Information
Explanation for why data not available: Raw data cannot be provided per National Health Insurance Service (NHIS)(data provider)'s privacy policy.
